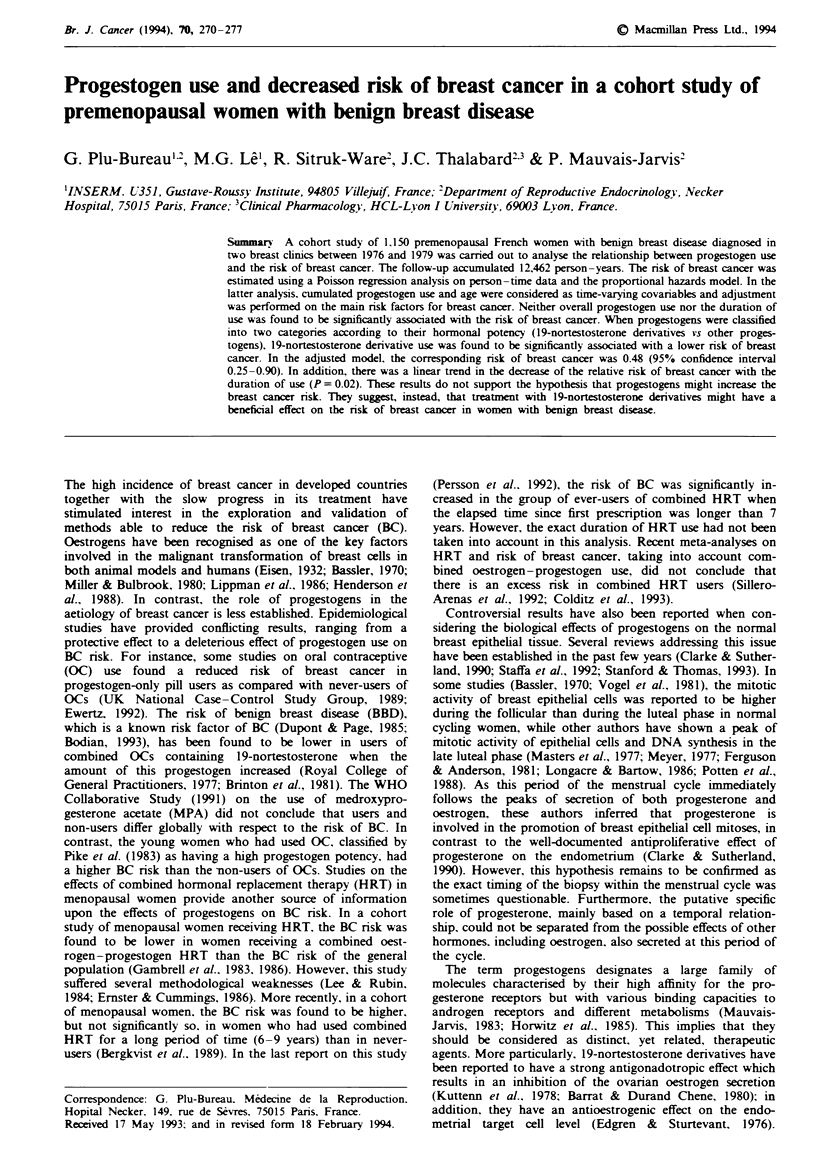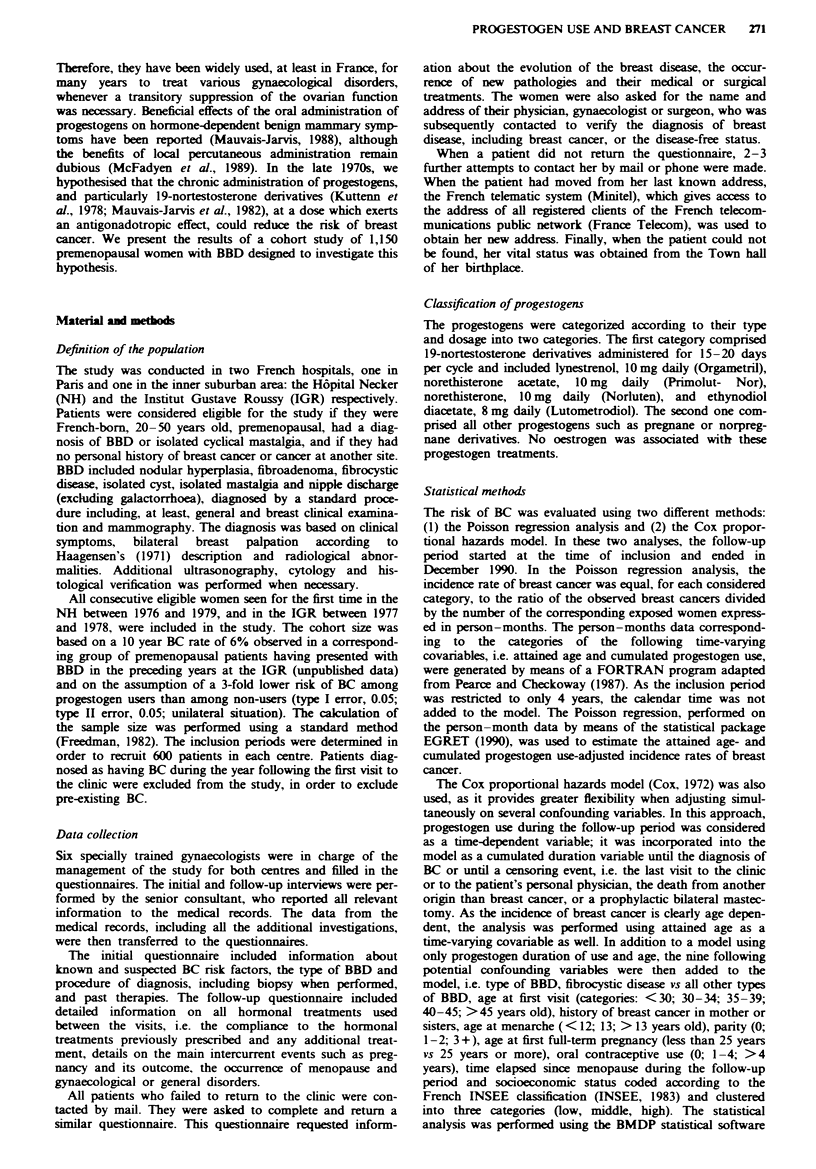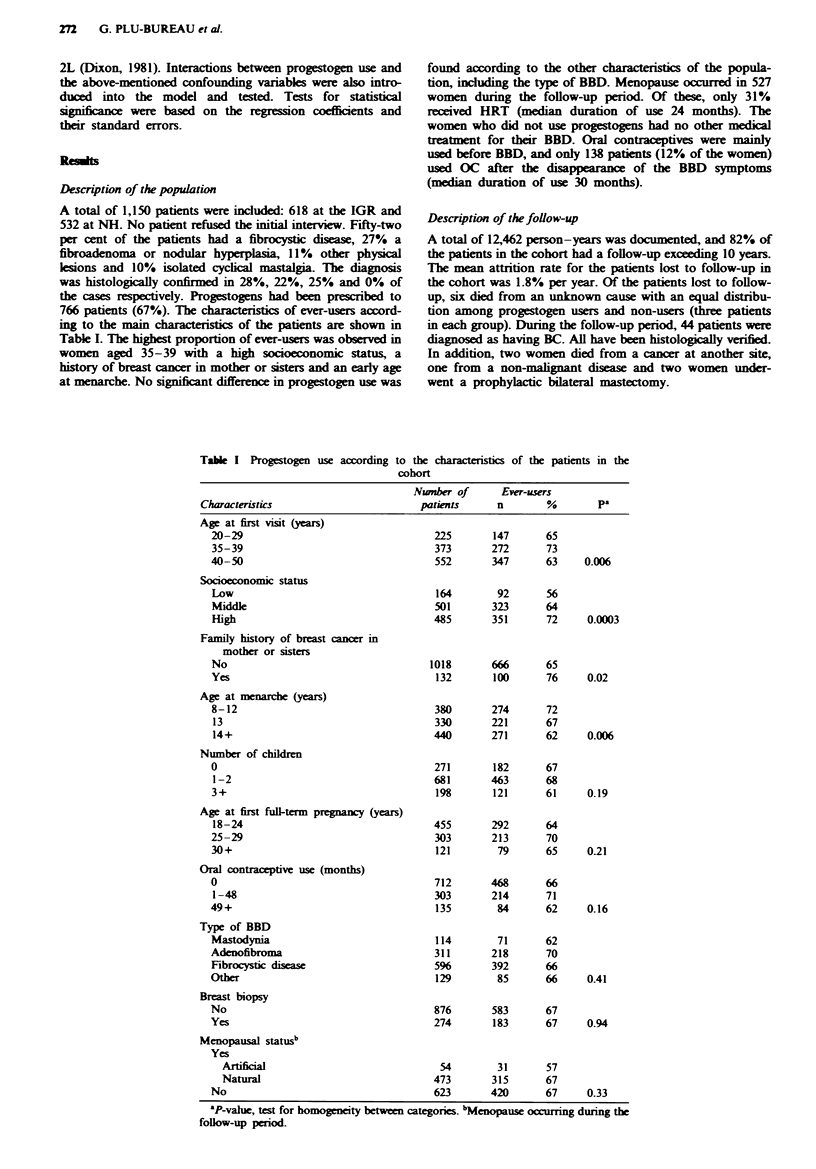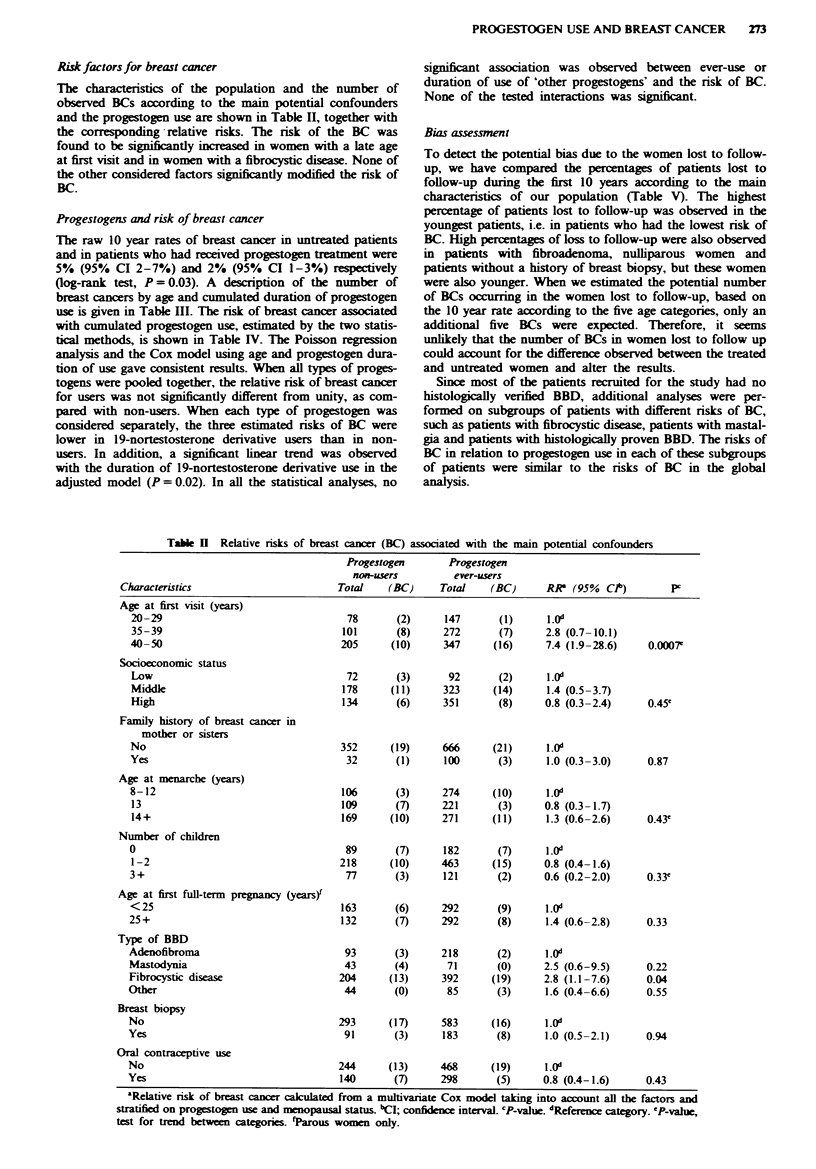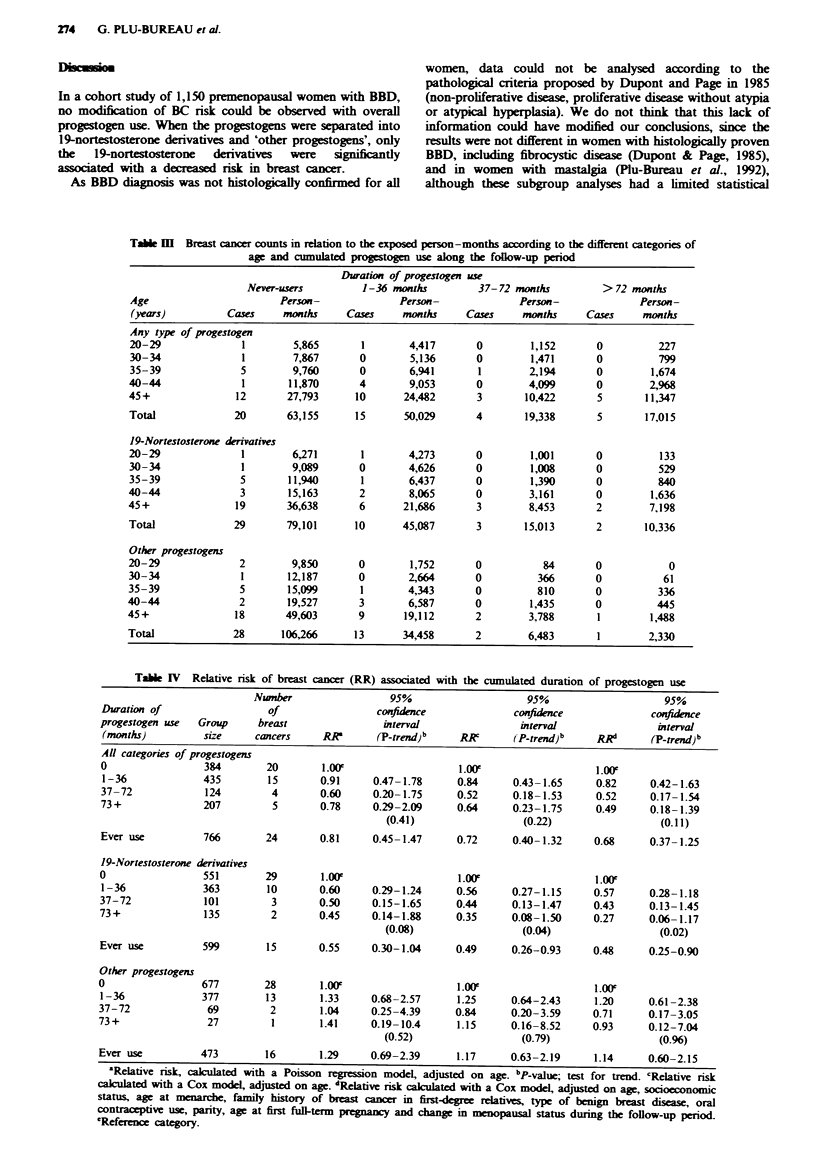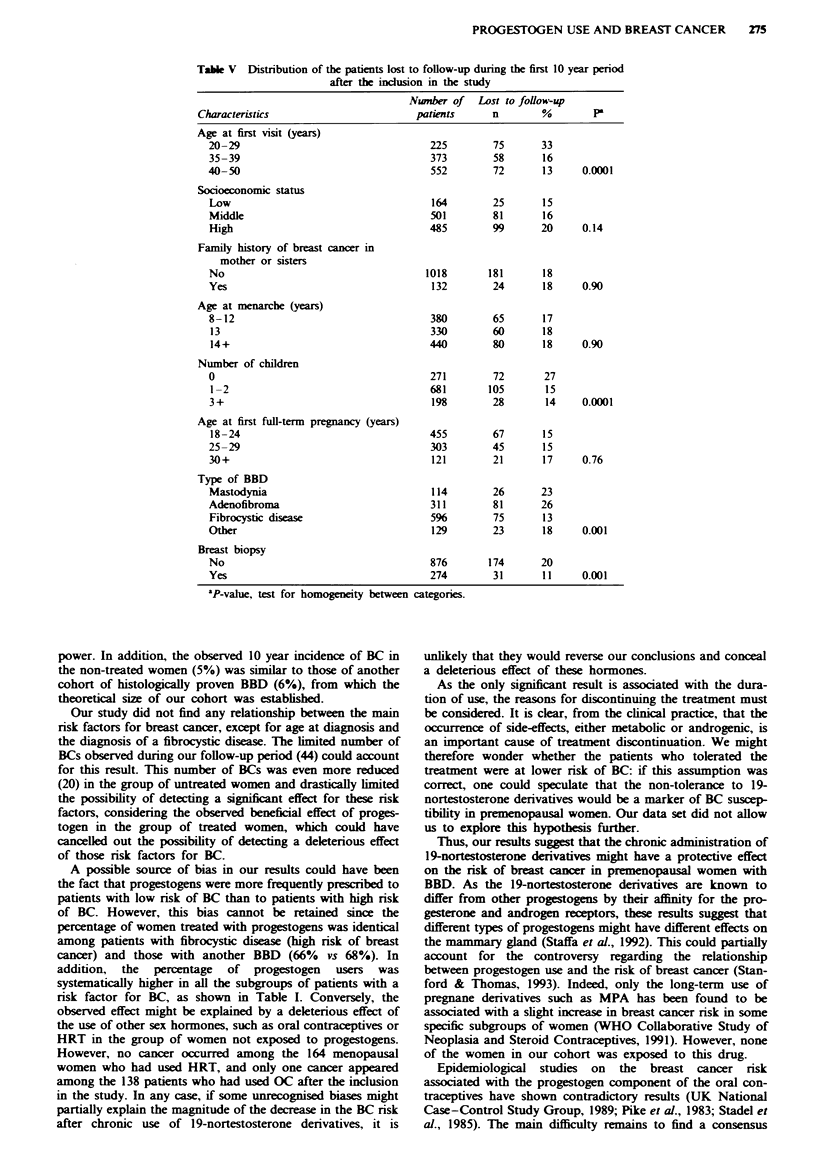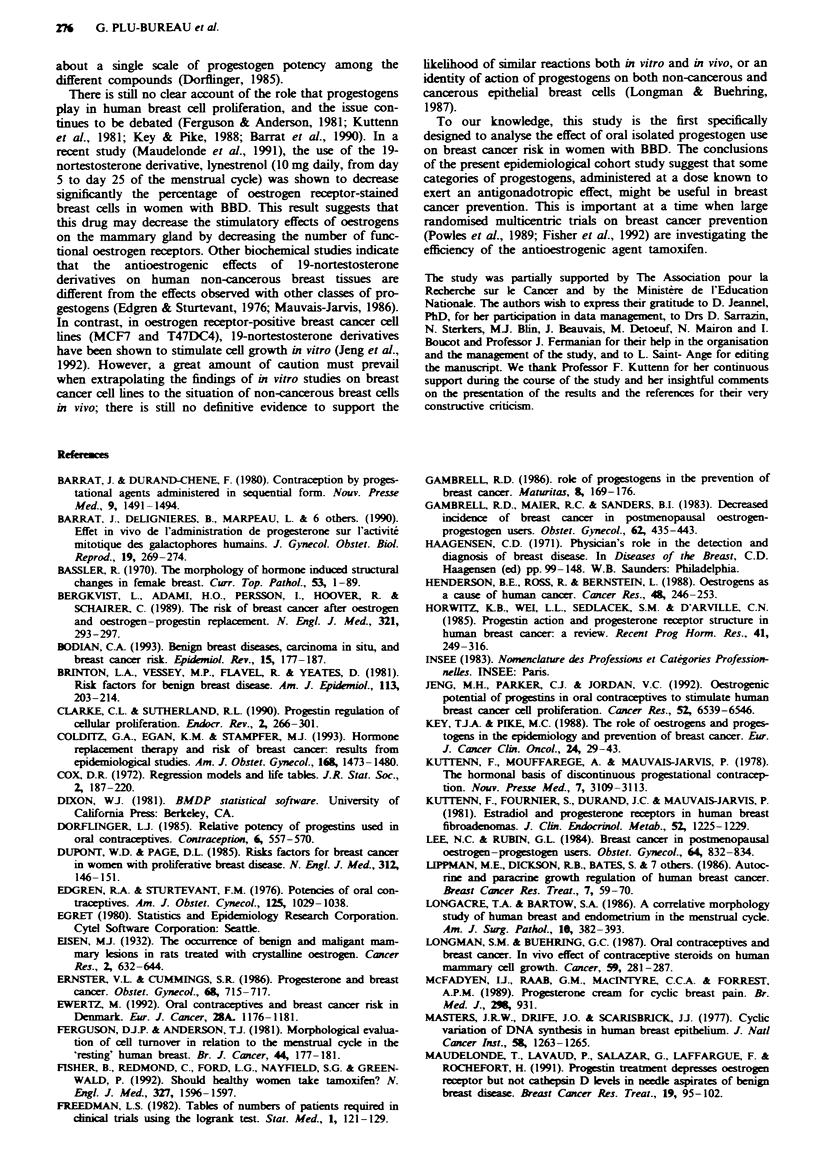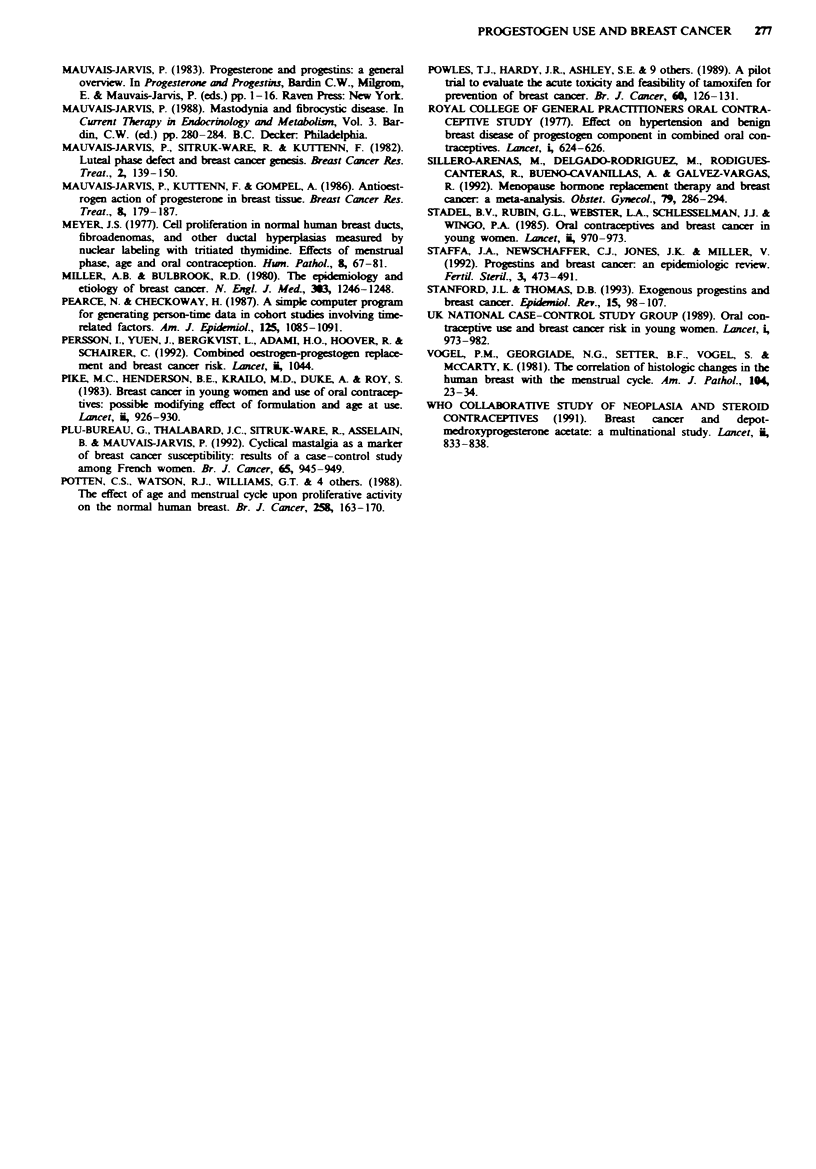# Progestogen use and decreased risk of breast cancer in a cohort study of premenopausal women with benign breast disease.

**DOI:** 10.1038/bjc.1994.291

**Published:** 1994-08

**Authors:** G. Plu-Bureau, M. G. Lê, R. Sitruk-Ware, J. C. Thalabard, P. Mauvais-Jarvis

**Affiliations:** INSERM. U351, Gustave-Roussy Institute, Villejuif, France.

## Abstract

A cohort study of 1,150 premenopausal French women with benign breast disease diagnosed in two breast clinics between 1976 and 1979 was carried out to analyse the relationship between progestogen use and the risk of breast cancer. The follow-up accumulated 12,462 person-years. The risk of breast cancer was estimated using a Poisson regression analysis on person-time data and the proportional hazards model. In the latter analysis, cumulated progestogen use and age were considered as time-varying covariables and adjustment was performed on the main risk factors for breast cancer. Neither overall progestogen use nor the duration of use was found to be significantly associated with the risk of breast cancer. When progestogens were classified into two categories according to their hormonal potency (19-nortestosterone derivatives vs other progestogens), 19-nortestosterone derivative use was found to be significantly associated with a lower risk of breast cancer. In the adjusted model, the corresponding risk of breast cancer was 0.48 (95% confidence interval 0.25-0.90). In addition, there was a linear trend in the decrease of the relative risk of breast cancer with the duration of use (P = 0.02). These results do not support the hypothesis that progestogens might increase the breast cancer risk. They suggest, instead, that treatment with 19-nortestosterone derivatives might have a beneficial effect on the risk of breast cancer in women with benign breast disease.


Br. J. Cancer (1994), 70, 270-277 © Macmillan Press Ltd., 1994

### Progestogen use and decreased risk of breast cancer in a cohort study of premenopausal women with benign breast disease

G. Plu-Bureau[1,2], M.G. Lê[1], R. Sitruk-Ware[2], J.C. Thalabard[2,3] & P. Mauvais-Jarvis[2]

[1]*INSERM. U351, Gustave-Roussy Institute, 94805 Villejuif, France;* [2]*Department of Reproductive Endocrinology, Necker Hospital, 75015 Paris, France;* [3]*Clinical Pharmacology, HCL-Lyon I University, 69003 Lyon, France.*

**Summary** A cohort study of 1,150 premenopausal French women with benign breast disease diagnosed in two breast clinics between 1976 and 1979 was carried out to analyse the relationship between progestogen use and the risk of breast cancer. The follow-up accumulated 12,462 person-years. The risk of breast cancer was estimated using a Poisson regression analysis on person-time data and the proportional hazards model. In the latter analysis, cumulated progestogen use and age were considered as time-varying covariables and adjustment was performed on the main risk factors for breast cancer. Neither overall progestogen use nor the duration of use was found to be significantly associated with the risk of breast cancer. When progestogens were classified into two categories according to their hormonal potency (19-nortestosterone derivatives *vs* other progestogens), 19-nortestosterone derivative use was found to be significantly associated with a lower risk of breast cancer. In the adjusted model, the corresponding risk of breast cancer was 0.48 (95% confidence interval 0.25-0.90). In addition, there was a linear trend in the decrease of the relative risk of breast cancer with the duration of use ($P = 0.02$). These results do not support the hypothesis that progestogens might increase the breast cancer risk. They suggest, instead, that treatment with 19-nortestosterone derivatives might have a beneficial effect on the risk of breast cancer in women with benign breast disease.

The high incidence of breast cancer in developed countries together with the slow progress in its treatment have stimulated interest in the exploration and validation of methods able to reduce the risk of breast cancer (BC). Oestrogens have been recognised as one of the key factors involved in the malignant transformation of breast cells in both animal models and humans (Eisen, 1932; Bassler, 1970; Miller & Bulbrook, 1980; Lippman *et al.*, 1986; Henderson *et al.*, 1988). In contrast, the role of progestogens in the aetiology of breast cancer is less established. Epidemiological studies have provided conflicting results, ranging from a protective effect to a deleterious effect of progestogen use on BC risk. For instance, some studies on oral contraceptive (OC) use found a reduced risk of breast cancer in progestogen-only pill users as compared with never-users of OCs (UK National Case-Control Study Group, 1989; Ewertz, 1992). The risk of benign breast disease (BBD), which is a known risk factor of BC (Dupont & Page, 1985; Bodian, 1993), has been found to be lower in users of combined OCs containing 19-nortestosterone when the amount of this progestogen increased (Royal College of General Practitioners, 1977; Brinton *et al.*, 1981). The WHO Collaborative Study (1991) on the use of medroxyprogesterone acetate (MPA) did not conclude that users and non-users differ globally with respect to the risk of BC. In contrast, the young women who had used OC, classified by Pike *et al.* (1983) as having a high progestogen potency, had a higher BC risk than the non-users of OCs. Studies on the effects of combined hormonal replacement therapy (HRT) in menopausal women provide another source of information upon the effects of progestogens on BC risk. In a cohort study of menopausal women receiving HRT, the BC risk was found to be lower in women receiving a combined oestrogen-progestogen HRT than the BC risk of the general population (Gambrell *et al.*, 1983, 1986). However, this study suffered several methodological weaknesses (Lee & Rubin, 1984; Ernster & Cummings, 1986). More recently, in a cohort of menopausal women, the BC risk was found to be higher, but not significantly so, in women who had used combined HRT for a long period of time (6-9 years) than in never-users (Bergkvist *et al.*, 1989). In the last report on this study (Persson *et al.*, 1992), the risk of BC was significantly increased in the group of ever-users of combined HRT when the elapsed time since first prescription was longer than 7 years. However, the exact duration of HRT use had not been taken into account in this analysis. Recent meta-analyses on HRT and risk of breast cancer, taking into account combined oestrogen-progestogen use, did not conclude that there is an excess risk in combined HRT users (Sillero-Arenas *et al.*, 1992; Colditz *et al.*, 1993).

Controversial results have also been reported when considering the biological effects of progestogens on the normal breast epithelial tissue. Several reviews addressing this issue have been established in the past few years (Clarke & Sutherland, 1990; Staffa *et al.*, 1992; Stanford & Thomas, 1993). In some studies (Bassler, 1970; Vogel *et al.*, 1981), the mitotic activity of breast epithelial cells was reported to be higher during the follicular than during the luteal phase in normal cycling women, while other authors have shown a peak of mitotic activity of epithelial cells and DNA synthesis in the late luteal phase (Masters *et al.*, 1977; Meyer, 1977; Ferguson & Anderson, 1981; Longacre & Bartow, 1986; Potten *et al.*, 1988). As this period of the menstrual cycle immediately follows the peaks of secretion of both progesterone and oestrogen, these authors inferred that progesterone is involved in the promotion of breast epithelial cell mitoses, in contrast to the well-documented antiproliferative effect of progesterone on the endometrium (Clarke & Sutherland, 1990). However, this hypothesis remains to be confirmed as the exact timing of the biopsy within the menstrual cycle was sometimes questionable. Furthermore, the putative specific role of progesterone, mainly based on a temporal relationship, could not be separated from the possible effects of other hormones, including oestrogen, also secreted at this period of the cycle.

The term progestogens designates a large family of molecules characterised by their high affinity for the progesterone receptors but with various binding capacities to androgen receptors and different metabolisms (Mauvais-Jarvis, 1983; Horwitz *et al.*, 1985). This implies that they should be considered as distinct, yet related, therapeutic agents. More particularly, 19-nortestosterone derivatives have been reported to have a strong antigonadotropic effect which results in an inhibition of the ovarian oestrogen secretion (Kuttenn *et al.*, 1978; Barrat & Durand Chene, 1980); in addition, they have an antioestrogenic effect on the endometrial target cell level (Edgren & Sturtevant, 1976).

Correspondence: G. Plu-Bureau, Médecine de la Reproduction, Hopital Necker, 149, rue de Sèvres, 75015 Paris, France.
Received 17 May 1993; and in revised form 18 February 1994.

Therefore, they have been widely used, at least in France, for many years to treat various gynaecological disorders, whenever a transitory suppression of the ovarian function was necessary. Beneficial effects of the oral administration of progestogens on hormone-dependent benign mammary symptoms have been reported (Mauvais-Jarvis, 1988), although the benefits of local percutaneous administration remain dubious (McFadyen *et al.*, 1989). In the late 1970s, we hypothesised that the chronic administration of progestogens, and particularly 19-nortestosterone derivatives (Kuttenn *et al.*, 1978; Mauvais-Jarvis *et al.*, 1982), at a dose which exerts an antigonadotropic effect, could reduce the risk of breast cancer. We present the results of a cohort study of 1,150 premenopausal women with BBD designed to investigate this hypothesis.

### Material and methods

### *Definition of the population*

The study was conducted in two French hospitals, one in Paris and one in the inner suburban area: the Hôpital Necker (NH) and the Institut Gustave Roussy (IGR) respectively. Patients were considered eligible for the study if they were French-born, 20–50 years old, premenopausal, had a diagnosis of BBD or isolated cyclical mastalgia, and if they had no personal history of breast cancer or cancer at another site. BBD included nodular hyperplasia, fibroadenoma, fibrocystic disease, isolated cyst, isolated mastalgia and nipple discharge (excluding galactorrhoea), diagnosed by a standard procedure including, at least, general and breast clinical examination and mammography. The diagnosis was based on clinical symptoms, bilateral breast palpation according to Haagensen's (1971) description and radiological abnormalities. Additional ultrasonography, cytology and histological verification was performed when necessary.

All consecutive eligible women seen for the first time in the NH between 1976 and 1979, and in the IGR between 1977 and 1978, were included in the study. The cohort size was based on a 10 year BC rate of 6% observed in a corresponding group of premenopausal patients having presented with BBD in the preceding years at the IGR (unpublished data) and on the assumption of a 3-fold lower risk of BC among progestogen users than among non-users (type I error, 0.05; type II error, 0.05; unilateral situation). The calculation of the sample size was performed using a standard method (Freedman, 1982). The inclusion periods were determined in order to recruit 600 patients in each centre. Patients diagnosed as having BC during the year following the first visit to the clinic were excluded from the study, in order to exclude pre-existing BC.

### *Data collection*

Six specially trained gynaecologists were in charge of the management of the study for both centres and filled in the questionnaires. The initial and follow-up interviews were performed by the senior consultant, who reported all relevant information to the medical records. The data from the medical records, including all the additional investigations, were then transferred to the questionnaires.

The initial questionnaire included information about known and suspected BC risk factors, the type of BBD and procedure of diagnosis, including biopsy when performed, and past therapies. The follow-up questionnaire included detailed information on all hormonal treatments used between the visits, i.e. the compliance to the hormonal treatments previously prescribed and any additional treatment, details on the main intercurrent events such as pregnancy and its outcome, the occurrence of menopause and gynaecological or general disorders.

All patients who failed to return to the clinic were contacted by mail. They were asked to complete and return a similar questionnaire. This questionnaire requested information about the evolution of the breast disease, the occurrence of new pathologies and their medical or surgical treatments. The women were also asked for the name and address of their physician, gynaecologist or surgeon, who was subsequently contacted to verify the diagnosis of breast disease, including breast cancer, or the disease-free status.

When a patient did not return the questionnaire, 2–3 further attempts to contact her by mail or phone were made. When the patient had moved from her last known address, the French telematic system (Minitel), which gives access to the address of all registered clients of the French telecommunications public network (France Telecom), was used to obtain her new address. Finally, when the patient could not be found, her vital status was obtained from the Town hall of her birthplace.

### *Classification of progestogens*

The progestogens were categorized according to their type and dosage into two categories. The first category comprised 19-nortestosterone derivatives administered for 15–20 days per cycle and included lynestrenol, 10 mg daily (Orgametril), norethisterone acetate, 10 mg daily (Primolut- Nor), norethisterone, 10 mg daily (Norluten), and ethynodiol diacetate, 8 mg daily (Lutometrodiol). The second one comprised all other progestogens such as pregnane or norpregnane derivatives. No oestrogen was associated with these progestogen treatments.

### *Statistical methods*

The risk of BC was evaluated using two different methods: (1) the Poisson regression analysis and (2) the Cox proportional hazards model. In these two analyses, the follow-up period started at the time of inclusion and ended in December 1990. In the Poisson regression analysis, the incidence rate of breast cancer was equal, for each considered category, to the ratio of the observed breast cancers divided by the number of the corresponding exposed women expressed in person–months. The person–months data corresponding to the categories of the following time-varying covariables, i.e. attained age and cumulated progestogen use, were generated by means of a FORTRAN program adapted from Pearce and Checkoway (1987). As the inclusion period was restricted to only 4 years, the calendar time was not added to the model. The Poisson regression, performed on the person–month data by means of the statistical package EGRET (1990), was used to estimate the attained age- and cumulated progestogen use-adjusted incidence rates of breast cancer.

The Cox proportional hazards model (Cox, 1972) was also used, as it provides greater flexibility when adjusting simultaneously on several confounding variables. In this approach, progestogen use during the follow-up period was considered as a time-dependent variable; it was incorporated into the model as a cumulated duration variable until the diagnosis of BC or until a censoring event, i.e. the last visit to the clinic or to the patient's personal physician, the death from another origin than breast cancer, or a prophylactic bilateral mastectomy. As the incidence of breast cancer is clearly age dependent, the analysis was performed using attained age as a time-varying covariable as well. In addition to a model using only progestogen duration of use and age, the nine following potential confounding variables were then added to the model, i.e. type of BBD, fibrocystic disease *vs* all other types of BBD, age at first visit (categories: $<30$; 30–34; 35–39; 40–45; $>45$ years old), history of breast cancer in mother or sisters, age at menarche ($<12$; 13; $>13$ years old), parity (0; 1–2; 3+), age at first full-term pregnancy (less than 25 years *vs* 25 years or more), oral contraceptive use (0; 1–4; $>4$ years), time elapsed since menopause during the follow-up period and socioeconomic status coded according to the French INSEE classification (INSEE, 1983) and clustered into three categories (low, middle, high). The statistical analysis was performed using the BMDP statistical software

2L (Dixon, 1981). Interactions between progestogen use and the above-mentioned confounding variables were also introduced into the model and tested. Tests for statistical significance were based on the regression coefficients and their standard errors.

### Results

### Description of the population

A total of 1,150 patients were included: 618 at the IGR and 532 at NH. No patient refused the initial interview. Fifty-two per cent of the patients had a fibrocystic disease, 27% a fibroadenoma or nodular hyperplasia, 11% other physical lesions and 10% isolated cyclical mastalgia. The diagnosis was histologically confirmed in 28%, 22%, 25% and 0% of the cases respectively. Progestogens had been prescribed to 766 patients (67%). The characteristics of ever-users according to the main characteristics of the patients are shown in Table I. The highest proportion of ever-users was observed in women aged 35–39 with a high socioeconomic status, a history of breast cancer in mother or sisters and an early age at menarche. No significant difference in progestogen use was found according to the other characteristics of the population, including the type of BBD. Menopause occurred in 527 women during the follow-up period. Of these, only 31% received HRT (median duration of use 24 months). The women who did not use progestogens had no other medical treatment for their BBD. Oral contraceptives were mainly used before BBD, and only 138 patients (12% of the women) used OC after the disappearance of the BBD symptoms (median duration of use 30 months).

### Description of the follow-up

A total of 12,462 person–years was documented, and 82% of the patients in the cohort had a follow-up exceeding 10 years. The mean attrition rate for the patients lost to follow-up in the cohort was 1.8% per year. Of the patients lost to follow-up, six died from an unknown cause with an equal distribution among progestogen users and non-users (three patients in each group). During the follow-up period, 44 patients were diagnosed as having BC. All have been histologically verified. In addition, two women died from a cancer at another site, one from a non-malignant disease and two women underwent a prophylactic bilateral mastectomy.

**Table I** Progestogen use according to the characteristics of the patients in the cohort

| *Characteristics* | *Number of patients* | *Ever-users* n | *Ever-users* % | P[a] |
|---|---|---|---|---|
| Age at first visit (years) | | | | |
| 20–29 | 225 | 147 | 65 | |
| 35–39 | 373 | 272 | 73 | |
| 40–50 | 552 | 347 | 63 | 0.006 |
| Socioeconomic status | | | | |
| Low | 164 | 92 | 56 | |
| Middle | 501 | 323 | 64 | |
| High | 485 | 351 | 72 | 0.0003 |
| Family history of breast cancer in mother or sisters | | | | |
| No | 1018 | 666 | 65 | |
| Yes | 132 | 100 | 76 | 0.02 |
| Age at menarche (years) | | | | |
| 8–12 | 380 | 274 | 72 | |
| 13 | 330 | 221 | 67 | |
| 14+ | 440 | 271 | 62 | 0.006 |
| Number of children | | | | |
| 0 | 271 | 182 | 67 | |
| 1–2 | 681 | 463 | 68 | |
| 3+ | 198 | 121 | 61 | 0.19 |
| Age at first full-term pregnancy (years) | | | | |
| 18–24 | 455 | 292 | 64 | |
| 25–29 | 303 | 213 | 70 | |
| 30+ | 121 | 79 | 65 | 0.21 |
| Oral contraceptive use (months) | | | | |
| 0 | 712 | 468 | 66 | |
| 1–48 | 303 | 214 | 71 | |
| 49+ | 135 | 84 | 62 | 0.16 |
| Type of BBD | | | | |
| Mastodynia | 114 | 71 | 62 | |
| Adenofibroma | 311 | 218 | 70 | |
| Fibrocystic disease | 596 | 392 | 66 | |
| Other | 129 | 85 | 66 | 0.41 |
| Breast biopsy | | | | |
| No | 876 | 583 | 67 | |
| Yes | 274 | 183 | 67 | 0.94 |
| Menopausal status[b] | | | | |
| Yes | | | | |
| Artificial | 54 | 31 | 57 | |
| Natural | 473 | 315 | 67 | |
| No | 623 | 420 | 67 | 0.33 |

[a]*P*-value, test for homogeneity between categories. [b]Menopause occurring during the follow-up period.

*Risk factors for breast cancer*

The characteristics of the population and the number of observed BCs according to the main potential confounders and the progestogen use are shown in Table II, together with the corresponding relative risks. The risk of the BC was found to be significantly increased in women with a late age at first visit and in women with a fibrocystic disease. None of the other considered factors significantly modified the risk of BC.

*Progestogens and risk of breast cancer*

The raw 10 year rates of breast cancer in untreated patients and in patients who had received progestogen treatment were 5% (95% CI 2–7%) and 2% (95% CI 1–3%) respectively (log-rank test, $P = 0.03$). A description of the number of breast cancers by age and cumulated duration of progestogen use is given in Table III. The risk of breast cancer associated with cumulated progestogen use, estimated by the two statistical methods, is shown in Table IV. The Poisson regression analysis and the Cox model using age and progestogen duration of use gave consistent results. When all types of progestogens were pooled together, the relative risk of breast cancer for users was not significantly different from unity, as compared with non-users. When each type of progestogen was considered separately, the three estimated risks of BC were lower in 19-nortestosterone derivative users than in non-users. In addition, a significant linear trend was observed with the duration of 19-nortestosterone derivative use in the adjusted model ($P = 0.02$). In all the statistical analyses, no significant association was observed between ever-use or duration of use of 'other progestogens' and the risk of BC. None of the tested interactions was significant.

*Bias assessment*

To detect the potential bias due to the women lost to follow-up, we have compared the percentages of patients lost to follow-up during the first 10 years according to the main characteristics of our population (Table V). The highest percentage of patients lost to follow-up was observed in the youngest patients, i.e. in patients who had the lowest risk of BC. High percentages of loss to follow-up were also observed in patients with fibroadenoma, nulliparous women and patients without a history of breast biopsy, but these women were also younger. When we estimated the potential number of BCs occurring in the women lost to follow-up, based on the 10 year rate according to the five age categories, only an additional five BCs were expected. Therefore, it seems unlikely that the number of BCs in women lost to follow up could account for the difference observed between the treated and untreated women and alter the results.

Since most of the patients recruited for the study had no histologically verified BBD, additional analyses were performed on subgroups of patients with different risks of BC, such as patients with fibrocystic disease, patients with mastalgia and patients with histologically proven BBD. The risks of BC in relation to progestogen use in each of these subgroups of patients were similar to the risks of BC in the global analysis.

**Table II** Relative risks of breast cancer (BC) associated with the main potential confounders

| Characteristics | *Progestogen non-users* Total | (BC) | *Progestogen ever-users* Total | (BC) | RR[a] (95% CI[b]) | P[c] |
|---|---|---|---|---|---|---|
| Age at first visit (years) | | | | | | |
| 20–29 | 78 | (2) | 147 | (1) | 1.0[d] | |
| 35–39 | 101 | (8) | 272 | (7) | 2.8 (0.7–10.1) | |
| 40–50 | 205 | (10) | 347 | (16) | 7.4 (1.9–28.6) | 0.0007[e] |
| Socioeconomic status | | | | | | |
| Low | 72 | (3) | 92 | (2) | 1.0[d] | |
| Middle | 178 | (11) | 323 | (14) | 1.4 (0.5–3.7) | |
| High | 134 | (6) | 351 | (8) | 0.8 (0.3–2.4) | 0.45[e] |
| Family history of breast cancer in mother or sisters | | | | | | |
| No | 352 | (19) | 666 | (21) | 1.0[d] | |
| Yes | 32 | (1) | 100 | (3) | 1.0 (0.3–3.0) | 0.87 |
| Age at menarche (years) | | | | | | |
| 8–12 | 106 | (3) | 274 | (10) | 1.0[d] | |
| 13 | 109 | (7) | 221 | (3) | 0.8 (0.3–1.7) | |
| 14+ | 169 | (10) | 271 | (11) | 1.3 (0.6–2.6) | 0.43[e] |
| Number of children | | | | | | |
| 0 | 89 | (7) | 182 | (7) | 1.0[d] | |
| 1–2 | 218 | (10) | 463 | (15) | 0.8 (0.4–1.6) | |
| 3+ | 77 | (3) | 121 | (2) | 0.6 (0.2–2.0) | 0.33[e] |
| Age at first full-term pregnancy (years)[f] | | | | | | |
| <25 | 163 | (6) | 292 | (9) | 1.0[d] | |
| 25+ | 132 | (7) | 292 | (8) | 1.4 (0.6–2.8) | 0.33 |
| Type of BBD | | | | | | |
| Adenofibroma | 93 | (3) | 218 | (2) | 1.0[d] | |
| Mastodynia | 43 | (4) | 71 | (0) | 2.5 (0.6–9.5) | 0.22 |
| Fibrocystic disease | 204 | (13) | 392 | (19) | 2.8 (1.1–7.6) | 0.04 |
| Other | 44 | (0) | 85 | (3) | 1.6 (0.4–6.6) | 0.55 |
| Breast biopsy | | | | | | |
| No | 293 | (17) | 583 | (16) | 1.0[d] | |
| Yes | 91 | (3) | 183 | (8) | 1.0 (0.5–2.1) | 0.94 |
| Oral contraceptive use | | | | | | |
| No | 244 | (13) | 468 | (19) | 1.0[d] | |
| Yes | 140 | (7) | 298 | (5) | 0.8 (0.4–1.6) | 0.43 |

[a]Relative risk of breast cancer calculated from a multivariate Cox model taking into account all the factors and stratified on progestogen use and menopausal status. [b]CI; confidence interval. [c]*P*-value. [d]Reference category. [e]*P*-value, test for trend between categories. [f]Parous women only.

### Discussion

In a cohort study of 1,150 premenopausal women with BBD, no modification of BC risk could be observed with overall progestogen use. When the progestogens were separated into 19-nortestosterone derivatives and 'other progestogens', only the 19-nortestosterone derivatives were significantly associated with a decreased risk in breast cancer.

As BBD diagnosis was not histologically confirmed for all women, data could not be analysed according to the pathological criteria proposed by Dupont and Page in 1985 (non-proliferative disease, proliferative disease without atypia or atypical hyperplasia). We do not think that this lack of information could have modified our conclusions, since the results were not different in women with histologically proven BBD, including fibrocystic disease (Dupont & Page, 1985), and in women with mastalgia (Plu-Bureau *et al.*, 1992), although these subgroup analyses had a limited statistical

**Table III** Breast cancer counts in relation to the exposed person-months according to the different categories of age and cumulated progestogen use along the follow-up period

| | Duration of progestogen use | | | | | | | |
|---|---|---|---|---|---|---|---|---|
| | *Never-users* | | *1–36 months* | | *37–72 months* | | *>72 months* | |
| *Age (years)* | *Cases* | *Person-months* | *Cases* | *Person-months* | *Cases* | *Person-months* | *Cases* | *Person-months* |
| *Any type of progestogen* | | | | | | | | |
| 20–29 | 1 | 5,865 | 1 | 4,417 | 0 | 1,152 | 0 | 227 |
| 30–34 | 1 | 7,867 | 0 | 5,136 | 0 | 1,471 | 0 | 799 |
| 35–39 | 5 | 9,760 | 0 | 6,941 | 1 | 2,194 | 0 | 1,674 |
| 40–44 | 1 | 11,870 | 4 | 9,053 | 0 | 4,099 | 0 | 2,968 |
| 45+ | 12 | 27,793 | 10 | 24,482 | 3 | 10,422 | 5 | 11,347 |
| Total | 20 | 63,155 | 15 | 50,029 | 4 | 19,338 | 5 | 17,015 |
| *19-Nortestosterone derivatives* | | | | | | | | |
| 20–29 | 1 | 6,271 | 1 | 4,273 | 0 | 1,001 | 0 | 133 |
| 30–34 | 1 | 9,089 | 0 | 4,626 | 0 | 1,008 | 0 | 529 |
| 35–39 | 5 | 11,940 | 1 | 6,437 | 0 | 1,390 | 0 | 840 |
| 40–44 | 3 | 15,163 | 2 | 8,065 | 0 | 3,161 | 0 | 1,636 |
| 45+ | 19 | 36,638 | 6 | 21,686 | 3 | 8,453 | 2 | 7,198 |
| Total | 29 | 79,101 | 10 | 45,087 | 3 | 15,013 | 2 | 10,336 |
| *Other progestogens* | | | | | | | | |
| 20–29 | 2 | 9,850 | 0 | 1,752 | 0 | 84 | 0 | 0 |
| 30–34 | 1 | 12,187 | 0 | 2,664 | 0 | 366 | 0 | 61 |
| 35–39 | 5 | 15,099 | 1 | 4,343 | 0 | 810 | 0 | 336 |
| 40–44 | 2 | 19,527 | 3 | 6,587 | 0 | 1,435 | 0 | 445 |
| 45+ | 18 | 49,603 | 9 | 19,112 | 2 | 3,788 | 1 | 1,488 |
| Total | 28 | 106,266 | 13 | 34,458 | 2 | 6,483 | 1 | 2,330 |

**Table IV** Relative risk of breast cancer (RR) associated with the cumulated duration of progestogen use

| *Duration of progestogen use (months)* | *Group size* | *Number of breast cancers* | *RR*[a] | *95% confidence interval (P-trend)*[b] | *RR*[c] | *95% confidence interval (P-trend)*[b] | *RR*[d] | *95% confidence interval (P-trend)*[b] |
|---|---|---|---|---|---|---|---|---|
| *All categories of progestogens* | | | | | | | | |
| 0 | 384 | 20 | 1.00[e] | | 1.00[e] | | 1.00[e] | |
| 1–36 | 435 | 15 | 0.91 | 0.47–1.78 | 0.84 | 0.43–1.65 | 0.82 | 0.42–1.63 |
| 37–72 | 124 | 4 | 0.60 | 0.20–1.75 | 0.52 | 0.18–1.53 | 0.52 | 0.17–1.54 |
| 73+ | 207 | 5 | 0.78 | 0.29–2.09 (0.41) | 0.64 | 0.23–1.75 (0.22) | 0.49 | 0.18–1.39 (0.11) |
| Ever use | 766 | 24 | 0.81 | 0.45–1.47 | 0.72 | 0.40–1.32 | 0.68 | 0.37–1.25 |
| *19-Nortestosterone derivatives* | | | | | | | | |
| 0 | 551 | 29 | 1.00[e] | | 1.00[e] | | 1.00[e] | |
| 1–36 | 363 | 10 | 0.60 | 0.29–1.24 | 0.56 | 0.27–1.15 | 0.57 | 0.28–1.18 |
| 37–72 | 101 | 3 | 0.50 | 0.15–1.65 | 0.44 | 0.13–1.47 | 0.43 | 0.13–1.45 |
| 73+ | 135 | 2 | 0.45 | 0.14–1.88 (0.08) | 0.35 | 0.08–1.50 (0.04) | 0.27 | 0.06–1.17 (0.02) |
| Ever use | 599 | 15 | 0.55 | 0.30–1.04 | 0.49 | 0.26–0.93 | 0.48 | 0.25–0.90 |
| *Other progestogens* | | | | | | | | |
| 0 | 677 | 28 | 1.00[e] | | 1.00[e] | | 1.00[e] | |
| 1–36 | 377 | 13 | 1.33 | 0.68–2.57 | 1.25 | 0.64–2.43 | 1.20 | 0.61–2.38 |
| 37–72 | 69 | 2 | 1.04 | 0.25–4.39 | 0.84 | 0.20–3.59 | 0.71 | 0.17–3.05 |
| 73+ | 27 | 1 | 1.41 | 0.19–10.4 (0.52) | 1.15 | 0.16–8.52 (0.79) | 0.93 | 0.12–7.04 (0.96) |
| Ever use | 473 | 16 | 1.29 | 0.69–2.39 | 1.17 | 0.63–2.19 | 1.14 | 0.60–2.15 |

[a]Relative risk, calculated with a Poisson regression model, adjusted on age. [b]*P*-value; test for trend. [c]Relative risk calculated with a Cox model, adjusted on age. [d]Relative risk calculated with a Cox model, adjusted on age, socioeconomic status, age at menarche, family history of breast cancer in first-degree relatives, type of benign breast disease, oral contraceptive use, parity, age at first full-term pregnancy and change in menopausal status during the follow-up period. [e]Reference category.

**Table V** Distribution of the patients lost to follow-up during the first 10 year period after the inclusion in the study

| *Characteristics* | *Number of patients* | *Lost to follow-up* n | % | P[a] |
|---|---|---|---|---|
| Age at first visit (years) | | | | |
| 20–29 | 225 | 75 | 33 | |
| 35–39 | 373 | 58 | 16 | |
| 40–50 | 552 | 72 | 13 | 0.0001 |
| Socioeconomic status | | | | |
| Low | 164 | 25 | 15 | |
| Middle | 501 | 81 | 16 | |
| High | 485 | 99 | 20 | 0.14 |
| Family history of breast cancer in mother or sisters | | | | |
| No | 1018 | 181 | 18 | |
| Yes | 132 | 24 | 18 | 0.90 |
| Age at menarche (years) | | | | |
| 8–12 | 380 | 65 | 17 | |
| 13 | 330 | 60 | 18 | |
| 14+ | 440 | 80 | 18 | 0.90 |
| Number of children | | | | |
| 0 | 271 | 72 | 27 | |
| 1–2 | 681 | 105 | 15 | |
| 3+ | 198 | 28 | 14 | 0.0001 |
| Age at first full-term pregnancy (years) | | | | |
| 18–24 | 455 | 67 | 15 | |
| 25–29 | 303 | 45 | 15 | |
| 30+ | 121 | 21 | 17 | 0.76 |
| Type of BBD | | | | |
| Mastodynia | 114 | 26 | 23 | |
| Adenofibroma | 311 | 81 | 26 | |
| Fibrocystic disease | 596 | 75 | 13 | |
| Other | 129 | 23 | 18 | 0.001 |
| Breast biopsy | | | | |
| No | 876 | 174 | 20 | |
| Yes | 274 | 31 | 11 | 0.001 |

[a]*P*-value, test for homogeneity between categories.

power. In addition, the observed 10 year incidence of BC in the non-treated women (5%) was similar to those of another cohort of histologically proven BBD (6%), from which the theoretical size of our cohort was established.

Our study did not find any relationship between the main risk factors for breast cancer, except for age at diagnosis and the diagnosis of a fibrocystic disease. The limited number of BCs observed during our follow-up period (44) could account for this result. This number of BCs was even more reduced (20) in the group of untreated women and drastically limited the possibility of detecting a significant effect for these risk factors, considering the observed beneficial effect of progestogen in the group of treated women, which could have cancelled out the possibility of detecting a deleterious effect of those risk factors for BC.

A possible source of bias in our results could have been the fact that progestogens were more frequently prescribed to patients with low risk of BC than to patients with high risk of BC. However, this bias cannot be retained since the percentage of women treated with progestogens was identical among patients with fibrocystic disease (high risk of breast cancer) and those with another BBD (66% *vs* 68%). In addition, the percentage of progestogen users was systematically higher in all the subgroups of patients with a risk factor for BC, as shown in Table I. Conversely, the observed effect might be explained by a deleterious effect of the use of other sex hormones, such as oral contraceptives or HRT in the group of women not exposed to progestogens. However, no cancer occurred among the 164 menopausal women who had used HRT, and only one cancer appeared among the 138 patients who had used OC after the inclusion in the study. In any case, if some unrecognised biases might partially explain the magnitude of the decrease in the BC risk after chronic use of 19-nortestosterone derivatives, it is unlikely that they would reverse our conclusions and conceal a deleterious effect of these hormones.

As the only significant result is associated with the duration of use, the reasons for discontinuing the treatment must be considered. It is clear, from the clinical practice, that the occurrence of side-effects, either metabolic or androgenic, is an important cause of treatment discontinuation. We might therefore wonder whether the patients who tolerated the treatment were at lower risk of BC: if this assumption was correct, one could speculate that the non-tolerance to 19-nortestosterone derivatives would be a marker of BC susceptibility in premenopausal women. Our data set did not allow us to explore this hypothesis further.

Thus, our results suggest that the chronic administration of 19-nortestosterone derivatives might have a protective effect on the risk of breast cancer in premenopausal women with BBD. As the 19-nortestosterone derivatives are known to differ from other progestogens by their affinity for the progesterone and androgen receptors, these results suggest that different types of progestogens might have different effects on the mammary gland (Staffa *et al.*, 1992). This could partially account for the controversy regarding the relationship between progestogen use and the risk of breast cancer (Stanford & Thomas, 1993). Indeed, only the long-term use of pregnane derivatives such as MPA has been found to be associated with a slight increase in breast cancer risk in some specific subgroups of women (WHO Collaborative Study of Neoplasia and Steroid Contraceptives, 1991). However, none of the women in our cohort was exposed to this drug.

Epidemiological studies on the breast cancer risk associated with the progestogen component of the oral contraceptives have shown contradictory results (UK National Case–Control Study Group, 1989; Pike *et al.*, 1983; Stadel *et al.*, 1985). The main difficulty remains to find a consensus about a single scale of progestogen potency among the different compounds (Dorflinger, 1985).

There is still no clear account of the role that progestogens play in human breast cell proliferation, and the issue continues to be debated (Ferguson & Anderson, 1981; Kuttenn *et al.*, 1981; Key & Pike, 1988; Barrat *et al.*, 1990). In a recent study (Maudelonde *et al.*, 1991), the use of the 19-nortestosterone derivative, lynestrenol (10 mg daily, from day 5 to day 25 of the menstrual cycle) was shown to decrease significantly the percentage of oestrogen receptor-stained breast cells in women with BBD. This result suggests that this drug may decrease the stimulatory effects of oestrogens on the mammary gland by decreasing the number of functional oestrogen receptors. Other biochemical studies indicate that the antioestrogenic effects of 19-nortestosterone derivatives on human non-cancerous breast tissues are different from the effects observed with other classes of progestogens (Edgren & Sturtevant, 1976; Mauvais-Jarvis, 1986). In contrast, in oestrogen receptor-positive breast cancer cell lines (MCF7 and T47DC4), 19-nortestosterone derivatives have been shown to stimulate cell growth *in vitro* (Jeng *et al.*, 1992). However, a great amount of caution must prevail when extrapolating the findings of *in vitro* studies on breast cancer cell lines to the situation of non-cancerous breast cells *in vivo*; there is still no definitive evidence to support the likelihood of similar reactions both *in vitro* and *in vivo*, or an identity of action of progestogens on both non-cancerous and cancerous epithelial breast cells (Longman & Buehring, 1987).

To our knowledge, this study is the first specifically designed to analyse the effect of oral isolated progestogen use on breast cancer risk in women with BBD. The conclusions of the present epidemiological cohort study suggest that some categories of progestogens, administered at a dose known to exert an antigonadotropic effect, might be useful in breast cancer prevention. This is important at a time when large randomised multicentric trials on breast cancer prevention (Powles *et al.*, 1989; Fisher *et al.*, 1992) are investigating the efficiency of the antioestrogenic agent tamoxifen.

The study was partially supported by The Association pour la Recherche sur le Cancer and by the Ministère de l'Education Nationale. The authors wish to express their gratitude to D. Jeannel, PhD, for her participation in data management, to Drs D. Sarrazin, N. Sterkers, M.J. Blin, J. Beauvais, M. Detoeuf, N. Mairon and I. Boucot and Professor J. Fermanian for their help in the organisation and the management of the study, and to L. Saint- Ange for editing the manuscript. We thank Professor F. Kuttenn for her continuous support during the course of the study and her insightful comments on the presentation of the results and the references for their very constructive criticism.

### References

BARRAT, J. & DURAND-CHENE, F. (1980). Contraception by progestational agents administered in sequential form. *Nouv. Presse Med.*, **9**, 1491–1494.

BARRAT, J., DELIGNIERES, B., MARPEAU, L. & 6 others. (1990). Effet in vivo de l'administration de progesterone sur l'activité mitotique des galactophores humains. *J. Gynecol. Obstet. Biol. Reprod.*, **19**, 269–274.

BASSLER, R. (1970). The morphology of hormone induced structural changes in female breast. *Curr. Top. Pathol.*, **53**, 1–89.

BERGKVIST, L., ADAMI, H.O., PERSSON, I., HOOVER, R. & SCHAIRER, C. (1989). The risk of breast cancer after oestrogen and oestrogen-progestin replacement. *N. Engl. J. Med.*, **321**, 293–297.

BODIAN, C.A. (1993). Benign breast diseases, carcinoma in situ, and breast cancer risk. *Epidemiol. Rev.*, **15**, 177–187.

BRINTON, L.A., VESSEY, M.P., FLAVEL, R. & YEATES, D. (1981). Risk factors for benign breast disease. *Am. J. Epidemiol.*, **113**, 203–214.

CLARKE, C.L. & SUTHERLAND, R.L. (1990). Progestin regulation of cellular proliferation. *Endocr. Rev.*, **2**, 266–301.

COLDITZ, G.A., EGAN, K.M. & STAMPFER, M.J. (1993). Hormone replacement therapy and risk of breast cancer: results from epidemiological studies. *Am. J. Obstet. Gynecol.*, **168**, 1473–1480.

COX, D.R. (1972). Regression models and life tables. *J.R. Stat. Soc.*, **2**, 187–220.

DIXON, W.J. (1981). *BMDP statistical software*. University of California Press: Berkeley, CA.

DORFLINGER, L.J. (1985). Relative potency of progestins used in oral contraceptives. *Contraception*, **6**, 557–570.

DUPONT, W.D. & PAGE, D.L. (1985). Risks factors for breast cancer in women with proliferative breast disease. *N. Engl. J. Med.*, **312**, 146–151.

EDGREN, R.A. & STURTEVANT, F.M. (1976). Potencies of oral contraceptives. *Am. J. Obstet. Cynecol.*, **125**, 1029–1038.

EGRET (1980). Statistics and Epidemiology Research Corporation. Cytel Software Corporation: Seattle.

EISEN, M.J. (1932). The occurrence of benign and maligant mammary lesions in rats treated with crystalline oestrogen. *Cancer Res.*, **2**, 632–644.

ERNSTER, V.L. & CUMMINGS, S.R. (1986). Progesterone and breast cancer. *Obstet. Gynecol.*, **68**, 715–717.

EWERTZ, M. (1992). Oral contraceptives and breast cancer risk in Denmark. *Eur. J. Cancer*, **28A**. 1176–1181.

FERGUSON, D.J.P. & ANDERSON, T.J. (1981). Morphological evaluation of cell turnover in relation to the menstrual cycle in the 'resting' human breast. *Br. J. Cancer*, **44**, 177–181.

FISHER, B., REDMOND, C., FORD, L.G., NAYFIELD, S.G. & GREENWALD, P. (1992). Should healthy women take tamoxifen? *N. Engl. J. Med.*, **327**, 1596–1597.

FREEDMAN, L.S. (1982). Tables of numbers of patients required in clinical trials using the logrank test. *Stat. Med.*, **1**, 121–129.

GAMBRELL, R.D. (1986). role of progestogens in the prevention of breast cancer. *Maturitas*, **8**, 169–176.

GAMBRELL, R.D., MAIER, R.C. & SANDERS, B.I. (1983). Decreased incidence of breast cancer in postmenopausal oestrogen-progestogen users. *Obstet. Gynecol.*, **62**, 435–443.

HAAGENSEN, C.D. (1971). Physician's role in the detection and diagnosis of breast disease. In *Diseases of the Breast*, C.D. Haagensen (ed) pp. 99–148. W.B. Saunders: Philadelphia.

HENDERSON, B.E., ROSS, R. & BERNSTEIN, L. (1988). Oestrogens as a cause of human cancer. *Cancer Res.*, **48**, 246–253.

HORWITZ, K.B., WEI, L.L., SEDLACEK, S.M. & D'ARVILLE, C.N. (1985). Progestin action and progesterone receptor structure in human breast cancer: a review. *Recent Prog Horm. Res.*, **41**, 249–316.

INSEE (1983). *Nomenclature des Professions et Catégories Professionnelles*. INSEE: Paris.

JENG, M.H., PARKER, C.J. & JORDAN, V.C. (1992). Oestrogenic potential of progestins in oral contraceptives to stimulate human breast cancer cell proliferation. *Cancer Res.*, **52**, 6539–6546.

KEY, T.J.A. & PIKE, M.C. (1988). The role of oestrogens and progestogens in the epidemiology and prevention of breast cancer. *Eur. J. Cancer Clin. Oncol.*, **24**, 29–43.

KUTTENN, F., MOUFFAREGE, A. & MAUVAIS-JARVIS, P. (1978). The hormonal basis of discontinuous progestational contraception. *Nouv. Presse Med.*, **7**, 3109–3113.

KUTTENN, F., FOURNIER, S., DURAND, J.C. & MAUVAIS-JARVIS, P. (1981). Estradiol and progesterone receptors in human breast fibroadenomas. *J. Clin. Endocrinol. Metab.*, **52**, 1225–1229.

LEE, N.C. & RUBIN, G.L. (1984). Breast cancer in postmenopausal oestrogen-progestogen users. *Obstet. Gynecol.*, **64**, 832–834.

LIPPMAN, M.E., DICKSON, R.B., BATES, S. & 7 others. (1986). Autocrine and paracrine growth regulation of human breast cancer. *Breast Cancer Res. Treat.*, **7**, 59–70.

LONGACRE, T.A. & BARTOW, S.A. (1986). A correlative morphology study of human breast and endometrium in the menstrual cycle. *Am. J. Surg. Pathol.*, **10**, 382–393.

LONGMAN, S.M. & BUEHRING, G.C. (1987). Oral contraceptives and breast cancer. In vivo effect of contraceptive steroids on human mammary cell growth. *Cancer*, **59**, 281–287.

MCFADYEN, I.J., RAAB, G.M., MACINTYRE, C.C.A. & FORREST, A.P.M. (1989). Progesterone cream for cyclic breast pain. *Br. Med. J.*, **298**, 931.

MASTERS, J.R.W., DRIFE, J.O. & SCARISBRICK, J.J. (1977). Cyclic variation of DNA synthesis in human breast epithelium. *J. Natl Cancer Inst.*, **58**, 1263–1265.

MAUDELONDE, T., LAVAUD, P., SALAZAR, G., LAFFARGUE, F. & ROCHEFORT, H. (1991). Progestin treatment depresses oestrogen receptor but not cathepsin D levels in needle aspirates of benign breast disease. *Breast Cancer Res. Treat.*, **19**, 95–102.

MAUVAIS-JARVIS, P. (1983). Progesterone and progestins: a general overview. In *Progesterone and Progestins*, Bardin C.W., Milgrom, E. & Mauvais-Jarvis, P. (eds.) pp. 1-16. Raven Press: New York.
MAUVAIS-JARVIS, P. (1988). Mastodynia and fibrocystic disease. In *Current Therapy in Endocrinology and Metabolism*, Vol. 3. Bardin, C.W. (ed.) pp. 280-284. B.C. Decker: Philadelphia.
MAUVAIS-JARVIS, P., SITRUK-WARE, R. & KUTTENN, F. (1982). Luteal phase defect and breast cancer genesis. *Breast Cancer Res. Treat.*, **2**, 139-150.
MAUVAIS-JARVIS, P., KUTTENN, F. & GOMPEL, A. (1986). Antioestrogen action of progesterone in breast tissue. *Breast Cancer Res. Treat.*, **8**, 179-187.
MEYER, J.S. (1977). Cell proliferation in normal human breast ducts, fibroadenomas, and other ductal hyperplasias measured by nuclear labeling with tritiated thymidine. Effects of menstrual phase, age and oral contraception. *Hum. Pathol.*, **8**, 67-81.
MILLER, A.B. & BULBROOK, R.D. (1980). The epidemiology and etiology of breast cancer. *N. Engl. J. Med.*, **303**, 1246-1248.
PEARCE, N. & CHECKOWAY, H. (1987). A simple computer program for generating person-time data in cohort studies involving time-related factors. *Am. J. Epidemiol.*, **125**, 1085-1091.
PERSSON, I., YUEN, J., BERGKVIST, L., ADAMI, H.O., HOOVER, R. & SCHAIRER, C. (1992). Combined oestrogen-progestogen replacement and breast cancer risk. *Lancet*, **ii**, 1044.
PIKE, M.C., HENDERSON, B.E., KRAILO, M.D., DUKE, A. & ROY, S. (1983). Breast cancer in young women and use of oral contraceptives: possible modifying effect of formulation and age at use. *Lancet*, **ii**, 926-930.
PLU-BUREAU, G., THALABARD, J.C., SITRUK-WARE, R., ASSELAIN, B. & MAUVAIS-JARVIS, P. (1992). Cyclical mastalgia as a marker of breast cancer susceptibility: results of a case-control study among French women. *Br. J. Cancer*, **65**, 945-949.
POTTEN, C.S., WATSON, R.J., WILLIAMS, G.T. & 4 others. (1988). The effect of age and menstrual cycle upon proliferative activity on the normal human breast. *Br. J. Cancer*, **258**, 163-170.
POWLES, T.J., HARDY, J.R., ASHLEY, S.E. & 9 others. (1989). A pilot trial to evaluate the acute toxicity and feasibility of tamoxifen for prevention of breast cancer. *Br. J. Cancer*, **60**, 126-131.
ROYAL COLLEGE OF GENERAL PRACTITIONERS ORAL CONTRACEPTIVE STUDY (1977). Effect on hypertension and benign breast disease of progestogen component in combined oral contraceptives. *Lancet*, **i**, 624-626.
SILLERO-ARENAS, M., DELGADO-RODRIGUEZ, M., RODIGUES-CANTERAS, R., BUENO-CAVANILLAS, A. & GALVEZ-VARGAS, R. (1992). Menopause hormone replacement therapy and breast cancer: a meta-analysis. *Obstet. Gynecol.*, **79**, 286-294.
STADEL, B.V., RUBIN, G.L., WEBSTER, L.A., SCHLESSELMAN, J.J. & WINGO, P.A. (1985). Oral contraceptives and breast cancer in young women. *Lancet*, **ii**, 970-973.
STAFFA, J.A., NEWSCHAFFER, C.J., JONES, J.K. & MILLER, V. (1992). Progestins and breast cancer: an epidemiologic review. *Fertil. Steril.*, **3**, 473-491.
STANFORD, J.L. & THOMAS, D.B. (1993). Exogenous progestins and breast cancer. *Epidemiol. Rev.*, **15**, 98-107.
UK NATIONAL CASE-CONTROL STUDY GROUP (1989). Oral contraceptive use and breast cancer risk in young women. *Lancet*, **i**, 973-982.
VOGEL, P.M., GEORGIADE, N.G., SETTER, B.F., VOGEL, S. & McCARTY, K. (1981). The correlation of histologic changes in the human breast with the menstrual cycle. *Am. J. Pathol.*, **104**, 23-34.
WHO COLLABORATIVE STUDY OF NEOPLASIA AND STEROID CONTRACEPTIVES (1991). Breast cancer and depot-medroxyprogesterone acetate: a multinational study. *Lancet*, **ii**, 833-838.